# Effects of COVID-19 home confinement on musculoskeletal health and physical performance in a cross-sectional study: results of the COVID-19 era online survey

**DOI:** 10.11604/pamj.2022.43.82.29068

**Published:** 2022-10-14

**Authors:** Mustapha Mouilly, Ahmed Omar Touhami Ahami, Bachir El Bouhali

**Affiliations:** 1 Department of Biology, Faculty of Sciences and Technics, University Moulay Ismail, Errachidia, Morocco,; 2 Department of Biology, Faculty of Sciences, University Ibn Tofail, Kenitra, Morocco

**Keywords:** COVID-19, musculoskeletal disorders, pandemic, physical activity, public health

## Abstract

**Introduction:**

the global pandemic of COVID-19 is the unique health crisis, the populations were exposed to situations of unprecedented confinement, this represents a major public health challenge, with a high risk of developing musculoskeletal disorders. The objective of the present work is to identify the potential effects of sedentary behavior on musculoskeletal health and physical performance.

**Methods:**

after two months of confinement a survey was uploaded and shared on Google’s online survey platform. Two research laboratories, University Moulay Ismail, University Ibn Tofail, promoted the survey which was developed on the basis of two questionnaires: the French version of the Nordic questionnaire musculoskeletal disorders and the French version of the Global Physical Activity Questionnaire (GPAQ).

**Results:**

out of 384 respondents, 209 (54.4%) were females 124 (32.3%) were between 30-49 years old (6.8%) and 123 (32%) had an underweight and overweight levels, respectively. One hundred and thirty-nine 139 (36.2%) reported sitting more than eight hours. The results of our survey show that many of our respondents had developed some sort of musculoskeletal pain during the confinement. One hundred and sixty two (42.2%) reported to have pain in the lower back region, 108 (28.1%) of participants reported to have pain in the neck region, 93 (28.2%) in the shoulder region, 97 (25.3) - in the upper back region and more women than men reported musculoskeletal pain in more than five regions. The results of our survey also show that 322 (83,9%) of the participants, after two months of confinement, were unable to do high intensity exercises for at least 10 min per day in their usual daytime activity, the prevalence of musculoskeletal pain differed across categories of body mass index (BMI) between males and females, (p<0.05).

**Conclusion:**

the results of this study show the existence of musculoskeletal disorders and deterioration in physical performance and strongly recommend the urgent implementation of prevention and remediation interventions.

## Introduction

The COVID-19 pandemic started in China in December 2019 and then reached Morocco in early March 2020. The absence of therapeutic medical intervention for COVID-19 infection has led to the recommendation that populations have to limit their social interactions and live at home for several weeks to several months. Although it is effective for infection control, this approach forces people to adopt unusual sedentary behaviors. Confinement provides boredom and social isolation. Prolonged immobilization is among the known risk factors for mental health which has been widely described in research [1-3]. The occurrence of musculoskeletal pain, particularly from the back, neck, shoulders, and head, which has increased in recent decades, has also been recounted in other studies with considerable effects. Dittmer *et al*. reported that immobilization of the musculoskeletal system could lead to decreased muscle strength and atrophy, decreased endurance, and osteoporosis [4]. This can affect the person´s ability to move and perform daily activities [5]. No study in Morocco has been conducted to determine the consequences of confinement in the era of COVID-19 on the musculoskeletal system and physical performance. And also, my experience and my initial training in the field of physiotherapy and rehabilitation have allowed me to discover by observation the prevalence of the musculoskeletal disorder in a number of negligible confined people; this pushed me to really reflect in consultation with my co-authors on this research topic.

Fundamentally, the available data shows that a few days/weeks of inactivity impair the O_2_ pathway at all levels, from the cardiovascular system, including peripheral circulation, to the oxidative function of skeletal muscles [6]. The situation of confinement, requiring some people a reduction in their professional activity and a limitation of personal life around daily routines at home can consecutively reduce their physical activity. Confinement can provoke rapid deterioration in cardiovascular health and consequently premature death among populations at cardiovascular risk because by confinement physical activity levels decrease and sedentary behavior increases [7]. The objective of the present work is to identify the potential effects of sedentary behavior on musculoskeletal health and physical performance during the COVID-19 outbreak.

## Methods

**Study design and setting:** a descriptive cross-sectional study was carried out after two months of confinement during the COVID-19 pandemic two weeks just before the date set for quarantine lift, by using an online survey, and explaining the purpose of the study. Respondents were assured of the confidentiality of their information.

**Study population:** a random sample of people aged 18-69 years were invited to participate, the final study sample consisted of 384 individuals. Participants must be between 18 and 69 years old and had to complete the questionnaire online. Incomplete questionnaires were not included in the study.

**Data collection:** the survey was uploaded and shared on Google´s online survey platform. This study was designed by a group of multidisciplinary academics and scientists. Two research laboratories, University Moulay Ismail; Faculty of Science and Techniques, Errachidia (South-Est of Morocco), University Ibn Tofail, faculty of science, Kenitra (North -West of Morocco), promoted the survey which was initially developed in French and later translated into the Moroccan dialect. The translation has been internally validated with a double translation process (translation and retranslation). The survey was developed on the basis of two questionnaires: the French version of the Nordic questionnaire musculoskeletal disorders, validated and recognized as a reliable tool for epidemiological surveillance [8], and the French version of the Global Physical Activity Questionnaire (GPAQ), developed in 2002 by the World Health Organization as part of the Health Surveillance Program (STEPS) [9].

**Statistical analysis:** data entry and data analysis were performed using SPSS 15. The χ^2^ test was used to compare two categorical values with a significance level set at 5% and the Mann-Whitney test was used to test the difference between two quantitative variables with asymmetric distributions. Differences were considered significant if the p-value was < 0.05.

**Ethical considerations:** the authors have received the agreement of the respondents.

## Results

**Socio-demographic parameters:** out of 384 respondents, 209 (54.4%) were females and 175 (45.6%) were males. The majority of respondents, 213 (55.5%) were under the age of 30 years old while 124 (32.3%) were between 30-49 years old. 201 (52.3%) of respondents had the normal range of BMI, while 26 (6.8%) and 123 (32%) had underweight and overweight levels, respectively (Table 1). Out of 384 respondents, at least 322 (83%) respondents reported that they did not practice high-intensity exercises of 10mn minimum during the day. The most common position adopted during daily routine was sitting or sleeping, 139(36.2%) reported sitting more than eight hours and 161(41.9%) of respondents adopted the same position for less than eight hours while 84 (21.9%) had fewer than four hours in that position.

**Table 1:** patient characteristics (N=384)

Characteristics	Frequencies	Percentages
**Age**		
18 -29	213	(55.5) *
30- 49	124	(32.3) *
50 - 69	47	(12.2)
**Sex**		
Male	175	(45.6) *
Female	209	(54.4) *
**Body mass index (BMI) Kg/m^2^**		
Underweight (<18.5)	26	(6.8) *
Normal range (18.5-24.9)	201	(52.3) *
Overweight (25-29.9)	123	(32) *
Obese (≥ 30	34	(8.9) *
Right-handed	253	(91.9) *
left-handed	20	(5.2) *
Ambidextrous	11	(2.9) *
**High intensity exercises of 10mn**		
No	322	(83.9) *
Yes	62	(16.1) *
Number of days per week	0	(0; 0) **
**Exercises of moderate intensity of 10mn**		
No	260	(67.7) *
Yes	124	(32.3) *
Number of days per week	0	(0; 3) **
**Walking or cycling for 10mn**		
No	145	(37.8) *
Yes	239	(62.3) *
Number of days per week	3	(0; 5) **
**High intensity sport**		
No	239	(62.2) *
Yes	145	(37.8) *
Number of days per week	0	(0; 2) **
**Sitting/lying time**		
<4h	84	(21.9) *
Between 4h and 8h	161	(41.9) *
8h and more	139	(36.2) *

* Frequency (%) ; ** Median (range)

**Musculoskeletal disorders:** the results of our survey show that many of our respondents had developed some sort of musculoskeletal pain during the confinement. The distribution of the exact location of the musculoskeletal disorder pain is given in Table 2. Most of the respondents had inactivity-related pain in many regions. Out of 384 respondents, 108(28.1%) reported having pain in the neck region, 93(28.2%) in the shoulder region, 162(42.2%) reported having pain in the lower back region, 104(27.1%) - in the knee region, 97(25.3) - in the upper back region and 57(14.8%) ankle and feet. At least 35(9.1) also complained about other regions like the hand, fingers, and elbow. More women than men reported musculoskeletal pain in more than five regions (neck, shoulder, upper back, knee, hip, and ankle) for those participants who had developed musculoskeletal pain than for those who reported not having such pain, with statistically significant values (p<0.05) (Table 2). The majority of males had most pain complaints in the dorsal, lumbar, and cervical regions, however, the pain in the lower back region is equal in both sexes, (p=0.06).

**Table 2:** anatomical regions with musculoskeletal pains among population in the COVID-19 era during confinement (N=384)

Pain location	Respondents			P-value
	Females	Males	Total	
Neck				0.04*
Yes	68 (32.5)	40 (22.9)	108(28.1)	
No	141(67.5)	135(77.1)	276(71.9)	
Shoulder				0.04*
Yes	59(28.2)	34(19.4)	93(28.2)	
No	150(71.8)	141(80.6)	291(71.8)	
Elbow				0.86
Yes	25(12)	22(12.6)	47(12.2)	
No	184(88)	153(87.4)	337(87.8)	
Wrist and hand				0.46
Yes	33(15.8)	23(13.1)	56(14.6)	
No	176(84.2)	152(86.9)	328(85.4)	
Fingers				0.98
Yes	19(9.1)	16(9.1)	35(9.1)	
No	190(90.9)	159(90.9)	349(90.9)	
Upper back				0.03*
Yes	62(29.7)	35(20)	97(25.3)	
No	147(70.3)	140(80)	287(74.7)	
Lower back				0.06
Yes	97(46.4)	65(37.1)	162(42.2)	
No	112(53.6)	110(62.9)	222(57.8)	
Hip				0.04*
Yes	47(22.5)	25(14.3)	72(18.8)	
No	162(77.5)	150(85.7)	312(81.2)	
Knee				0.009*
Yes	68(32.5)	36(20.6)	104(27.1)	
No	141(67.5)	139(79.4)	280(72.9)	
Ankle and foot				0.001*
Yes	43(20.6)	14(8)	57(14.8)	
No	166(79.4)	161(92)	327(85.2)	

* Pearson Chi^2^ test

**Physical activity status:** the results of our survey also show that 322(83.9%) of the participants, after two months of confinement, were unable to do high-intensity exercises for at least 10 min per day in their usual daytime activity, this was equivalent for both sexes(p>0.05). Otherwise, 260(67.7%) participants were also unable to do moderate-intensity exercise during the day. in the same way, the sports activities are intense or moderate, the majority of the participants 239(62.2%) were unable to do it, and this was equal for the two sexes. Additionally, 145(37.8%) of respondents also reported that they were unable to walk or cycle for 10 minutes limited due to pain, especially in women, p<0.05. Out of 384 respondents, the prevalence of musculoskeletal pain differed across categories of BMI between males and females, (p<0.05). However, the study shows that the difference is not significant between the three groups of sedentary behavior when analyses were run separately for women and men, p>0.05 (Table 3). Our study also showed that there is a statistically significant difference between the two sexes with regard to, the number of days per week of moderate-intensity exercises, high-intensity sports, and the number of days of walking for 10 minutes, p<0.05 (Table 3).

**Table 3:** physical activity status among population in the COVID-19 era (N=384)

	Respondents			P-value
	Females	Males	Total	
**BMI (Kg/m^2^)**				0.04*
Underweight	16(7.7)	10(5.7)	26(6.8)	
Normal range	121(57.9)	80(45.7)	201(52.3)	
Overweight	56(26.8)	17(38.3)	123(32)	
Obese	16(7.7)	18(8.3)	34(8.9)	
**High intensity exercises of 10mn**				0.84*
Yes	33(15.8)	29(16.6)	62(16.1)	
No	176(84.2)	146(83.4)	322(83.9)	
**Exercises of moderate intensity of 10mn**				0.15*
Yes	74 (64.6)	50(28.6)	124(32.3)	
No	135 (35.4)	125(71.4)	260(67.7)	
**Walking or cycling for 10mn**				<0.001*
Yes	108(51.7)	131(74.9)	239(62.2)	
No	101(48.3)	44(25.1)	145(37.8)	
**High intensity sport**				<0.001*
Yes	59(28.2)	150(71.8)	145(37.8)	
No	89(50.9)	86(49.1)	239(62.2)	
**Moderate intensity sport**				0.01*
Yes	66(31.6)	77(44.0)	143(37.2)	
No	143(68.4)	98(56.0)	241(62.8)	
**Sitting/lying time**				0.4**
<4h	43(20.6)	41(23.4)	84(21.9)	
Between 4h and 8h	84 (40.2)	77(40)	161(41.9)	
8h and more	82(39.2)	57(32.6)	137(36.2)	
Number of days of high intensity	0(0;0)	0(0;0)	-	0.53**
Number of days of moderate intensity days	0(0;4)	0(0;2)	-	0.004**
Number of days of intense sports activity	0(0;1)	0(0;3)	-	0.000**
Number of days of moderate sports activity	0(0;3)	0(0;3)	-	0.13**
Number of days of walking or cycling for 10mn	2(0;5)	4(0;6)	-	0.000**

BMI: Body mass index; *frequency (%); **Median (range); *Pearson Chi^2^ test; **Mann Whitney test

## Discussion

The objective of this study is to identify the potential effects of sedentary behavior on musculoskeletal health and physical performance during the COVID-19 outbreak. This study was done on a sample of 384 members. To our knowledge, this is the first study of this kind to be done in Morocco. The prevalence of inactivity-related musculoskeletal disorders after two months with restricted social interactions and living in home confinement is found with a difference between the regions of the body. Out of 384 respondents, 162 (42.2%) participants reported that they were suffering from lower back pain at the time of the confinement, 108(28.1%) reported having pain in the neck region, 93 (28.2%) in the shoulder region, 104 (27.1%) - in the knee region, 97 (25.3) - in the upper back region and 57 (14.8%) ankle and feet. The prevalence of musculoskeletal disorders in women turns out to be higher compared to their male counterparts. Very limited activity was observed in our population during this pandemic COVID-19 which requires home confinement. Both sexes have limitations in daily exercises with a high and moderate volume of muscular contractions.

Our findings are similar to other such studies which investigated the association between sedentary behavior and musculoskeletal disorders [6,10]. Recent evidence shows that inactivity also damages the neuromuscular junction and muscle denervation [6], and the negative effect of inactivity on the musculoskeletal system has long been reported [11,12]. In another study when they compared the muscle biopsy of elderly sedentary people with that of other elderly people with a long history of high-level leisure sports, significantly much less denervated fibers are found in athletes [13], and in the same study, it was reported that routine exercise may protect against loss of motor units and muscle tissue [13].

Musculoskeletal disorders are described as a common problem in the working population in a sitting position especially if they take the wrong position for a long time, this evidence was reported by a study of musculoskeletal disorders among computer operators [14], however, a systematic review has found inconclusive evidence of a relationship between sedentary behavior and musculoskeletal disorders in adults [15]. On the other hand, by classifying a sedentary lifestyle at three levels; low, moderate, and high levels of sedentary behavior, a recent study has shown that there is an association between more hours of sedentary behavior and increased occurrence of musculoskeletal pain [16].

The prevalence of musculoskeletal disorders in women turns out to be higher compared to their male counterparts, this can be explained by their higher body weight, their smaller size as well as their differences in strength and muscle composition, this has also been found in other studies [17-19], especially the most exposed region is the spine. Similarly, in an Australian study, it was found that women have a higher risk of developing musculoskeletal disorders in the ankle [20]. A similar distribution between the sexes was observed in a systematic review [21].

The current period of social confinement is likely to have negative effects on many factors that influence physical performance quality and may be particularly stressful for mothers because people are exposed to an unprecedented situation of confinement with an unknown duration. This was reported in a very recent study [3]. Very limited activity was observed in our population during this pandemic COVID-19 which requires home confinement. Both sexes have limitations in the daily exercises with a high and moderate volume of muscular contractions, this could be explained by the pain and the presence of musculoskeletal disorders, as it could be explained also by ignorance; that the exercise is of vital importance in maintaining muscle strength. A recent study dealing with the subject of the impact of sedentarism due to the COVID-19 home confinement on neuromuscular, cardiovascular, and metabolic health, recommends daily exercises with slow, low/medium-intensity high-volume contractions to preserve neuromuscular health [6]. Loss of muscle mass can be greatly avoided if resistance exercise, in various forms, has been applied during bed rest periods, this has been confirmed in numerous studies [22-24].

It is therefore very clear that sedentary behavior can lead to predictable muscle loss, musculoskeletal disorders, and consequently to deterioration in physical performance. In view of the confinement conditions, our study was limited to an online survey, which overall limited the number of participants to 384, apart from the incomplete results which were eliminated.

## Conclusion

It was necessary to protect the health of citizens, by imposing the confinement strategy, in view of this COVID-19 pandemic. However, after two months of isolation, the results of this study show a prevalence of musculoskeletal disorders and deterioration in physical performance in a large part of the participants in the different age groups. It is highly desirable to conduct more in-depth studies on the various risk factors liable to damage musculoskeletal quality and physical activity in the face of sedentary behavior. Prevention strategies and remediation techniques must be developed to relieve suffering during this time of confinement.

### What is known about this topic

- Sedentary behavior can provoke rapid deterioration in cardiovascular health and consequently premature death among populations at cardiovascular risk;
- Prolonged immobilization is among known risk factors for mental health.

### What this study adds

- It is the first study on musculoskeletal disorders in the confined population during the COVID-19 era in Morocco;
- Sedentary behavior can lead to predictable muscle loss, musculoskeletal disorders and consequently to deterioration in physical performance;
- A very limited activity was observed in our population in this pandemic COVID-19 which requires the home confinement.
